# 7,11-Bis(4-methyl­phen­yl)-2,4,8,10-tetra­azaspiro­[5.5]undecane-1,3,5,9-tetra­one

**DOI:** 10.1107/S1600536808022113

**Published:** 2008-07-19

**Authors:** Ali Mohammad Astaraki, Ayoob Bazgir, Fereshteh Faraji

**Affiliations:** a Department of Chemistry, Islamic Azad University, Dorood Branch, Dorood 688173551, Iran; b Department of Chemistry, Faculty of Science, Karaj Branch, Karaj, Iran

## Abstract

In the mol­ecule of the title compound, C_21_H_20_N_4_O_4_, the two methyl­phenyl rings are oriented at a dihedral angle of 59.32 (4)°. The other two rings have flattened-boat conformations. In the crystal structure, inter­molecular N—H⋯O hydrogen bonds link the mol­ecules. There are C—H⋯π contacts between a methyl­phenyl ring and methyl and methine groups.

## Related literature

For general background, see: Pradhan *et al.* (2006 ▶); Useglio *et al.* (2006 ▶); Kazmierski *et al.* (2006 ▶). For bond-length data, see: Allen *et al.* (1987 ▶). For ring conformation puckering parameters, see: Cremer & Pople (1975 ▶).
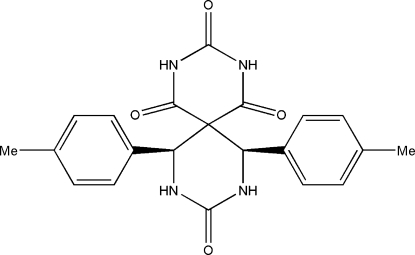


## Experimental

### 

#### Crystal data

C_21_H_20_N_4_O_4_
*M* *_r_* = 392.41
Monoclinic, 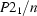

*a* = 8.852 (2) Å
*b* = 12.538 (3) Å
*c* = 17.259 (4) Å
β = 104.483 (18)°
*V* = 1854.6 (8) Å^3^
*Z* = 4
Mo *K*α radiation
μ = 0.1 mm^−1^
*T* = 298 (2) K
0.15 × 0.11 × 0.1 mm

#### Data collection

Stoe IPDSII diffractometer
Absorption correction: numerical (*X-SHAPE*; Stoe & Cie, 2005 ▶) *T*_min_ = 0.979, *T*_max_ = 0.991
17766 measured reflections
4456 independent reflections
3107 reflections with *I* > 2σ(*I*)
*R*_int_ = 0.093

#### Refinement

*R*[*F* ^2^ > 2σ(*F* ^2^)] = 0.081
*wR*(*F* ^2^) = 0.148
*S* = 1.13
4456 reflections
278 parameters
H atoms treated by a mixture of independent and constrained refinement
Δρ_max_ = 0.43 e Å^−3^
Δρ_min_ = −0.44 e Å^−3^

### 

Data collection: *X-AREA* (Stoe & Cie, 2005 ▶); cell refinement: *X-AREA*; data reduction: *X-RED*; program(s) used to solve structure: *SHELXS97* (Sheldrick, 2008 ▶); program(s) used to refine structure: *SHELXL97* (Sheldrick, 2008 ▶); molecular graphics: *ORTEP-3 for Windows* (Farrugia, 1997 ▶); software used to prepare material for publication: *WinGX* (Farrugia, 1999 ▶).

## Supplementary Material

Crystal structure: contains datablocks global, I. DOI: 10.1107/S1600536808022113/hk2496sup1.cif

Structure factors: contains datablocks I. DOI: 10.1107/S1600536808022113/hk2496Isup2.hkl

Additional supplementary materials:  crystallographic information; 3D view; checkCIF report

## Figures and Tables

**Table 1:** Hydrogen-bond geometry (Å, °) *Cg*4 is the centroid of the C11–C14/C16/C17 ring.

*D*—H⋯*A*	*D*—H	H⋯*A*	*D*⋯*A*	*D*—H⋯*A*
N1—H1*B*⋯O2^i^	0.80 (5)	2.39 (5)	3.004 (3)	135 (4)
N2—H2*B*⋯O3^ii^	0.77 (4)	2.32 (3)	3.065 (4)	164 (3)
N3—H3⋯O1^i^	0.82 (4)	2.54 (4)	3.181 (3)	136 (3)
N4—H4*D*⋯O1^iii^	0.87 (4)	1.92 (4)	2.785 (3)	174 (3)
C4—H4*B*⋯*Cg*4^iv^	0.96	3.02	3.721 (3)	131
C10—H10⋯*Cg*4^v^	0.98	3.11	3.914 (3)	141

Symmetry codes: (i) 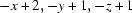; (ii) 
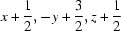
; (iii) 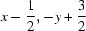, 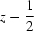; (iv) 
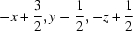
; (v) 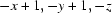.

**Fig. 1.**
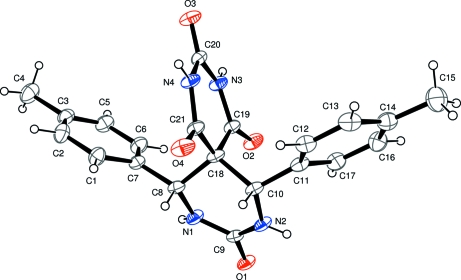
The molecular structure of the title molecule, with the atom-numbering scheme. Displacement ellipsoids are drawn at the 30% probability level.

**Fig. 2.**
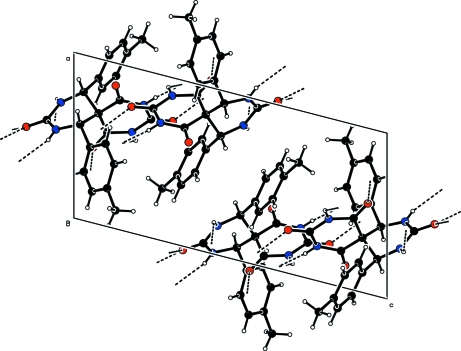
A partial packing diagram of the title compound. Hydrogen bonds are shown as dashed lines.

## supplementary crystallographic information

### Comment

Spiro compounds having cyclic structures fused at a central carbon are of recent interest, due to their interesting conformational feature and structural implications on biological systems (Pradhan *et al.*, 2006). The asymmetric characteristic of the molecule due to the chiral spiro carbon is one of the important criteria of the biological activities. For example, some spiro compounds show antibacterial and antiviral activities (Useglio *et al.*,  2006, Kazmierski *et al.*,  2006). We report herein the synthesis and crystal structure of the title compound.

In the molecule of the title compound (Fig. 1), the bond lengths (Allen *et al.*,  1987) and angles are within normal ranges. Ring A (C1-C3/C5-C7) and C (C11-C14/C16/C17) are, of course, planar and they are oriented at a dihedral angle of A/C = 59.32 (4)°. Rings B (N1/N2/C8-C10/C18) and D (N3/N4/C18-C21) have flattened-boat [φ = -54.22 (2)°, θ = 129.53 (3)° (for ring B) and φ = 52.72 (3)°, θ = 21.44 (3)° (for ring D)] conformations, having total puckering amplitudes, Q_T_, of 1.186 (3) and 0.174 (3) Å, respectively (Cremer & Pople, 1975).

In the crystal structure, intermolecular N-H···O hydrogen bonds (Table 1) link the molecules (Fig. 2), in which they may be effective in the stabilization of the structure. The C—H···π contacts (Table 1) between the ring C and the methyl and methine groups further stabilize the structure.

### Experimental

For the preparation of the title compound, a mixture of 4-methylbenzaldehyde (0.24 g, 2 mmol), barbituric acid (0.13 g, 1 mmol), and urea (0.06 g, 1 mmol) was heated at 373 K. After 2 h, the reaction mixture was washed with water (10 ml). The residue recrystallized from ethanol to afford the pure product (yield; 0.25 g, 65%, m.p. 519-521 K).

### Refinement

H1B, H2B, H3 and H4D atoms (for NH) were located in difference syntheses and refined isotropically [N-H = 0.77 (4)-0.87 (4) Å and U_iso_(H) = 0.032 (8) -0.076 (14) Å^2^]. The remaining H atoms were positioned geometrically, with C-H = 0.93, 0.98 and 0.96 Å for aromatic, methine and methyl H, respectively, and constrained to ride on their parent atoms with U_iso_(H) = 1.2U_eq_(C).

### Crystal data

**Table:**

C_21_H_20_N_4_O_4_	*F*_000_ = 824
*M**_r_* = 392.41	*D*_x_ = 1.405 Mg m^−^^3^
Monoclinic, *P*2_1_/*n*	Mo *K*α radiation λ = 0.71073 Å
Hall symbol: -P 2yn	Cell parameters from 2021 reflections
*a* = 8.852 (2) Å	θ = 2.0–28.1º
*b* = 12.538 (3) Å	µ = 0.1 mm^−^^1^
*c* = 17.259 (4) Å	*T* = 298 (2) K
β = 104.483 (18)º	Prism, yellow
*V* = 1854.6 (8) Å^3^	0.15 × 0.11 × 0.1 mm
*Z* = 4	

### Data collection

**Table:**

Stoe IPDSII diffractometer	*R*_int_ = 0.093
rotation method scans	θ_max_ = 28.1º
Absorption correction: Numericalshape of crystal determined optically (X-SHAPE; Stoe & Cie, 2005))	θ_min_ = 2.0º
*T*_min_ = 0.979, *T*_max_ = 0.991	*h* = −11→11
17766 measured reflections	*k* = −16→16
4456 independent reflections	*l* = −22→22
3107 reflections with *I* > 2σ(*I*)	

### Refinement

**Table:**

Refinement on *F*^2^	H atoms treated by a mixture of independent and constrained refinement
Least-squares matrix: full	*w* = 1/[σ^2^(*F*_o_^2^) + (0.0701*P*)^2^ + 0.8069*P*] where *P* = (*F*_o_^2^ + 2*F*_c_^2^)/3
*R*[*F*^2^ > 2σ(*F*^2^)] = 0.081	(Δ/σ)_max_ = 0.003
*wR*(*F*^2^) = 0.148	Δρ_max_ = 0.43 e Å^−^^3^
*S* = 1.13	Δρ_min_ = −0.44 e Å^−^^3^
4456 reflections	Extinction correction: none
278 parameters	

### Special details

**Table:**

Geometry. All e.s.d.'s (except the e.s.d. in the dihedral angle between two l.s. planes) are estimated using the full covariance matrix. The cell e.s.d.'s are taken into account individually in the estimation of e.s.d.'s in distances, angles and torsion angles; correlations between e.s.d.'s in cell parameters are only used when they are defined by crystal symmetry. An approximate (isotropic) treatment of cell e.s.d.'s is used for estimating e.s.d.'s involving l.s. planes.

### Fractional atomic coordinates and isotropic or equivalent isotropic displacement parameters (Å^2^)

**Table:**

	*x*	*y*	*z*	*U*_iso_*/*U*_eq_	
O1	1.0239 (3)	0.62989 (17)	0.65204 (12)	0.0446 (5)	
O2	1.0451 (3)	0.60240 (17)	0.43863 (13)	0.0463 (6)	
O3	0.7614 (3)	0.63921 (19)	0.18421 (13)	0.0522 (6)	
O4	0.6359 (3)	0.85414 (18)	0.36650 (13)	0.0520 (6)	
N1	0.8229 (3)	0.6195 (2)	0.54163 (15)	0.0417 (6)	
H1B	0.809 (5)	0.559 (4)	0.553 (3)	0.076 (14)*	
N2	0.9943 (3)	0.7595 (2)	0.55764 (14)	0.0403 (6)	
H2B	1.069 (4)	0.787 (3)	0.582 (2)	0.046 (10)*	
N3	0.8909 (3)	0.6150 (2)	0.31407 (15)	0.0393 (6)	
H3	0.936 (4)	0.563 (3)	0.3020 (19)	0.032 (8)*	
N4	0.7093 (3)	0.7516 (2)	0.27656 (14)	0.0381 (6)	
H4D	0.646 (4)	0.787 (3)	0.239 (2)	0.045 (9)*	
C1	0.4632 (4)	0.5999 (3)	0.3870 (2)	0.0453 (7)	
H1	0.4228	0.6665	0.3944	0.054*	
C2	0.3664 (4)	0.5232 (3)	0.3421 (2)	0.0516 (8)	
H2	0.2622	0.5397	0.3196	0.062*	
C3	0.4211 (4)	0.4227 (3)	0.33000 (18)	0.0455 (7)	
C4	0.3157 (5)	0.3384 (3)	0.2819 (2)	0.0634 (10)	
H4A	0.3123	0.2776	0.3152	0.076*	
H4B	0.3556	0.3172	0.2374	0.076*	
H4C	0.2125	0.3668	0.2626	0.076*	
C5	0.5765 (4)	0.4012 (3)	0.3651 (2)	0.0489 (8)	
H5	0.6162	0.334	0.3587	0.059*	
C6	0.6746 (4)	0.4773 (2)	0.40958 (19)	0.0448 (7)	
H6	0.7787	0.4606	0.432	0.054*	
C7	0.6191 (3)	0.5784 (2)	0.42101 (16)	0.0363 (6)	
C8	0.7243 (3)	0.6631 (2)	0.46862 (16)	0.0357 (6)	
H8	0.6568	0.7166	0.4842	0.043*	
C9	0.9505 (4)	0.6683 (2)	0.58658 (16)	0.0361 (6)	
C10	0.9253 (3)	0.8080 (2)	0.48001 (16)	0.0349 (6)	
H10	0.8511	0.8621	0.4885	0.042*	
C11	1.0480 (4)	0.8656 (2)	0.44822 (17)	0.0374 (7)	
C12	1.0027 (4)	0.9543 (2)	0.39888 (18)	0.0417 (7)	
H12	0.8984	0.9746	0.3844	0.05*	
C13	1.1118 (4)	1.0120 (2)	0.3715 (2)	0.0491 (8)	
H13	1.0792	1.0695	0.3374	0.059*	
C14	1.2688 (4)	0.9861 (2)	0.3938 (2)	0.0474 (8)	
C15	1.3894 (5)	1.0521 (3)	0.3674 (3)	0.0694 (11)	
H15A	1.3866	1.124	0.3862	0.083*	
H15B	1.3673	1.0521	0.3101	0.083*	
H15C	1.4911	1.0224	0.3892	0.083*	
C16	1.3123 (4)	0.8966 (3)	0.4421 (2)	0.0511 (8)	
H16	1.4168	0.8768	0.4568	0.061*	
C17	1.2051 (4)	0.8368 (3)	0.46843 (19)	0.0455 (8)	
H17	1.2375	0.7769	0.4999	0.055*	
C18	0.8296 (3)	0.7230 (2)	0.42113 (15)	0.0340 (6)	
C19	0.9342 (3)	0.6434 (2)	0.39333 (16)	0.0335 (6)	
C20	0.7848 (3)	0.6665 (2)	0.25345 (17)	0.0356 (6)	
C21	0.7178 (3)	0.7831 (2)	0.35308 (16)	0.0343 (6)	

### Atomic displacement parameters (Å^2^)

**Table:**

	*U*^11^	*U*^22^	*U*^33^	*U*^12^	*U*^13^	*U*^23^
O1	0.0555 (13)	0.0453 (11)	0.0249 (10)	−0.0030 (10)	−0.0049 (9)	0.0011 (8)
O2	0.0490 (13)	0.0443 (11)	0.0358 (11)	0.0170 (10)	−0.0075 (10)	−0.0017 (9)
O3	0.0634 (15)	0.0587 (14)	0.0279 (11)	0.0076 (12)	−0.0013 (10)	−0.0114 (10)
O4	0.0593 (14)	0.0517 (13)	0.0381 (12)	0.0261 (11)	−0.0007 (10)	−0.0024 (10)
N1	0.0491 (15)	0.0430 (14)	0.0264 (12)	−0.0046 (12)	−0.0030 (11)	0.0014 (10)
N2	0.0495 (15)	0.0402 (13)	0.0231 (11)	−0.0030 (12)	−0.0059 (11)	−0.0012 (10)
N3	0.0442 (14)	0.0381 (13)	0.0312 (13)	0.0117 (11)	0.0012 (10)	−0.0082 (10)
N4	0.0440 (14)	0.0405 (13)	0.0233 (11)	0.0111 (11)	−0.0040 (10)	0.0010 (10)
C1	0.0410 (16)	0.0415 (16)	0.0491 (19)	0.0038 (13)	0.0031 (14)	−0.0023 (13)
C2	0.0364 (16)	0.059 (2)	0.053 (2)	−0.0003 (15)	−0.0014 (14)	0.0024 (16)
C3	0.0520 (19)	0.0473 (17)	0.0335 (15)	−0.0100 (14)	0.0041 (14)	0.0023 (13)
C4	0.070 (2)	0.051 (2)	0.060 (2)	−0.0199 (18)	0.0006 (19)	−0.0005 (17)
C5	0.059 (2)	0.0394 (16)	0.0446 (18)	0.0011 (15)	0.0052 (15)	−0.0016 (13)
C6	0.0437 (17)	0.0436 (16)	0.0422 (17)	0.0069 (13)	0.0012 (14)	−0.0002 (13)
C7	0.0394 (15)	0.0402 (15)	0.0259 (13)	0.0013 (12)	0.0017 (11)	0.0023 (11)
C8	0.0411 (15)	0.0398 (14)	0.0231 (12)	0.0060 (12)	0.0023 (11)	−0.0020 (11)
C9	0.0441 (16)	0.0401 (14)	0.0214 (12)	0.0050 (12)	0.0034 (11)	−0.0023 (11)
C10	0.0435 (15)	0.0327 (13)	0.0233 (13)	0.0067 (12)	−0.0014 (11)	−0.0034 (10)
C11	0.0508 (17)	0.0291 (13)	0.0283 (13)	0.0034 (12)	0.0025 (12)	−0.0022 (11)
C12	0.0504 (18)	0.0349 (14)	0.0375 (16)	0.0116 (13)	0.0067 (13)	0.0021 (12)
C13	0.072 (2)	0.0317 (14)	0.0444 (18)	0.0090 (15)	0.0163 (16)	0.0030 (13)
C14	0.058 (2)	0.0382 (15)	0.0455 (18)	0.0030 (14)	0.0127 (15)	−0.0037 (13)
C15	0.074 (3)	0.054 (2)	0.081 (3)	−0.007 (2)	0.021 (2)	0.009 (2)
C16	0.0467 (18)	0.0494 (18)	0.053 (2)	0.0031 (15)	0.0053 (15)	0.0036 (15)
C17	0.0488 (18)	0.0423 (16)	0.0401 (17)	0.0082 (14)	0.0012 (14)	0.0065 (13)
C18	0.0415 (15)	0.0334 (13)	0.0221 (12)	0.0081 (12)	−0.0018 (11)	−0.0019 (10)
C19	0.0374 (15)	0.0328 (13)	0.0263 (13)	0.0054 (11)	0.0001 (11)	−0.0011 (10)
C20	0.0372 (15)	0.0376 (14)	0.0277 (13)	−0.0025 (12)	0.0002 (11)	−0.0015 (11)
C21	0.0367 (14)	0.0347 (13)	0.0265 (13)	0.0078 (12)	−0.0013 (11)	0.0006 (11)

### Geometric parameters (Å, °)

**Table:**

C1—C7	1.384 (4)	C11—C12	1.398 (4)
C1—C2	1.388 (5)	C12—C13	1.381 (5)
C1—H1	0.93	C12—H12	0.93
C2—C3	1.385 (5)	C13—C14	1.384 (5)
C2—H2	0.93	C13—H13	0.93
C3—C5	1.384 (5)	C14—C16	1.393 (5)
C3—C4	1.513 (5)	C14—C15	1.509 (5)
C4—H4A	0.96	C15—H15A	0.96
C4—H4B	0.96	C15—H15B	0.96
C4—H4C	0.96	C15—H15C	0.96
C5—C6	1.385 (4)	C16—C17	1.372 (5)
C5—H5	0.93	C16—H16	0.93
C6—C7	1.390 (4)	C17—H17	0.93
C6—H6	0.93	C18—C19	1.518 (4)
C7—C8	1.512 (4)	C18—C21	1.531 (4)
C8—N1	1.449 (4)	C19—O2	1.207 (3)
C8—C18	1.577 (4)	C19—N3	1.372 (3)
C8—H8	0.98	C20—O3	1.210 (3)
C9—O1	1.250 (3)	C20—N4	1.370 (4)
C9—N2	1.344 (4)	C20—N3	1.379 (4)
C9—N1	1.346 (4)	C21—O4	1.207 (3)
C10—N2	1.459 (3)	C21—N4	1.362 (4)
C10—C11	1.516 (4)	N1—H1B	0.80 (5)
C10—C18	1.568 (4)	N2—H2B	0.77 (4)
C10—H10	0.98	N3—H3	0.82 (3)
C11—C17	1.393 (4)	N4—H4D	0.87 (4)
			
C7—C1—C2	120.8 (3)	C12—C13—C14	121.4 (3)
C7—C1—H1	119.6	C12—C13—H13	119.3
C2—C1—H1	119.6	C14—C13—H13	119.3
C3—C2—C1	121.6 (3)	C13—C14—C16	117.6 (3)
C3—C2—H2	119.2	C13—C14—C15	121.6 (3)
C1—C2—H2	119.2	C16—C14—C15	120.8 (3)
C5—C3—C2	117.2 (3)	C14—C15—H15A	109.5
C5—C3—C4	120.9 (3)	C14—C15—H15B	109.5
C2—C3—C4	121.9 (3)	H15A—C15—H15B	109.5
C3—C4—H4A	109.5	C14—C15—H15C	109.5
C3—C4—H4B	109.5	H15A—C15—H15C	109.5
H4A—C4—H4B	109.5	H15B—C15—H15C	109.5
C3—C4—H4C	109.5	C17—C16—C14	121.8 (3)
H4A—C4—H4C	109.5	C17—C16—H16	119.1
H4B—C4—H4C	109.5	C14—C16—H16	119.1
C3—C5—C6	121.7 (3)	C16—C17—C11	120.3 (3)
C3—C5—H5	119.2	C16—C17—H17	119.8
C6—C5—H5	119.2	C11—C17—H17	119.8
C5—C6—C7	120.8 (3)	C19—C18—C21	114.1 (2)
C5—C6—H6	119.6	C19—C18—C10	112.2 (2)
C7—C6—H6	119.6	C21—C18—C10	107.7 (2)
C1—C7—C6	117.9 (3)	C19—C18—C8	109.7 (2)
C1—C7—C8	120.4 (3)	C21—C18—C8	106.3 (2)
C6—C7—C8	121.7 (3)	C10—C18—C8	106.3 (2)
N1—C8—C7	111.3 (2)	O2—C19—N3	121.0 (3)
N1—C8—C18	109.0 (2)	O2—C19—C18	122.5 (2)
C7—C8—C18	114.6 (2)	N3—C19—C18	116.5 (2)
N1—C8—H8	107.2	O3—C20—N4	122.2 (3)
C7—C8—H8	107.2	O3—C20—N3	122.1 (3)
C18—C8—H8	107.2	N4—C20—N3	115.7 (2)
O1—C9—N2	122.1 (3)	O4—C21—N4	120.6 (2)
O1—C9—N1	121.1 (3)	O4—C21—C18	121.3 (3)
N2—C9—N1	116.8 (3)	N4—C21—C18	118.0 (2)
N2—C10—C11	110.9 (2)	C9—N1—C8	124.5 (3)
N2—C10—C18	110.2 (2)	C9—N1—H1B	117 (3)
C11—C10—C18	114.1 (2)	C8—N1—H1B	118 (3)
N2—C10—H10	107	C9—N2—C10	127.1 (3)
C11—C10—H10	107	C9—N2—H2B	118 (3)
C18—C10—H10	107	C10—N2—H2B	114 (3)
C17—C11—C12	118.3 (3)	C19—N3—C20	127.0 (3)
C17—C11—C10	123.1 (3)	C19—N3—H3	115 (2)
C12—C11—C10	118.5 (3)	C20—N3—H3	118 (2)
C13—C12—C11	120.4 (3)	C21—N4—C20	126.5 (2)
C13—C12—H12	119.8	C21—N4—H4D	117 (2)
C11—C12—H12	119.8	C20—N4—H4D	117 (2)
			
C7—C1—C2—C3	0.4 (5)	N1—C8—C18—C19	65.8 (3)
C1—C2—C3—C5	0.5 (5)	C7—C8—C18—C19	−59.7 (3)
C1—C2—C3—C4	179.3 (4)	N1—C8—C18—C21	−170.3 (2)
C2—C3—C5—C6	−1.0 (5)	C7—C8—C18—C21	64.2 (3)
C4—C3—C5—C6	−179.8 (3)	N1—C8—C18—C10	−55.8 (3)
C3—C5—C6—C7	0.5 (5)	C7—C8—C18—C10	178.7 (2)
C2—C1—C7—C6	−0.8 (5)	C21—C18—C19—O2	167.7 (3)
C2—C1—C7—C8	178.9 (3)	C10—C18—C19—O2	44.8 (4)
C5—C6—C7—C1	0.4 (5)	C8—C18—C19—O2	−73.1 (3)
C5—C6—C7—C8	−179.4 (3)	C21—C18—C19—N3	−16.2 (4)
C1—C7—C8—N1	136.0 (3)	C10—C18—C19—N3	−139.0 (3)
C6—C7—C8—N1	−44.2 (4)	C8—C18—C19—N3	103.0 (3)
C1—C7—C8—C18	−99.7 (3)	C19—C18—C21—O4	−174.6 (3)
C6—C7—C8—C18	80.0 (3)	C10—C18—C21—O4	−49.3 (4)
N2—C10—C11—C17	27.2 (4)	C8—C18—C21—O4	64.3 (3)
C18—C10—C11—C17	−98.0 (3)	C19—C18—C21—N4	7.6 (4)
N2—C10—C11—C12	−150.1 (3)	C10—C18—C21—N4	132.9 (3)
C18—C10—C11—C12	84.6 (3)	C8—C18—C21—N4	−113.5 (3)
C17—C11—C12—C13	−0.4 (4)	O1—C9—N1—C8	174.7 (3)
C10—C11—C12—C13	177.0 (3)	N2—C9—N1—C8	−5.4 (5)
C11—C12—C13—C14	−1.9 (5)	C7—C8—N1—C9	164.5 (3)
C12—C13—C14—C16	2.8 (5)	C18—C8—N1—C9	37.2 (4)
C12—C13—C14—C15	−176.9 (3)	O1—C9—N2—C10	175.3 (3)
C13—C14—C16—C17	−1.4 (5)	N1—C9—N2—C10	−4.6 (5)
C15—C14—C16—C17	178.4 (4)	C11—C10—N2—C9	−146.8 (3)
C14—C16—C17—C11	−0.9 (5)	C18—C10—N2—C9	−19.4 (4)
C12—C11—C17—C16	1.8 (5)	O2—C19—N3—C20	−168.4 (3)
C10—C11—C17—C16	−175.5 (3)	C18—C19—N3—C20	15.4 (4)
N2—C10—C18—C19	−72.4 (3)	O3—C20—N3—C19	175.2 (3)
C11—C10—C18—C19	53.3 (3)	N4—C20—N3—C19	−3.9 (5)
N2—C10—C18—C21	161.2 (2)	O4—C21—N4—C20	−173.8 (3)
C11—C10—C18—C21	−73.2 (3)	C18—C21—N4—C20	4.0 (4)
N2—C10—C18—C8	47.6 (3)	O3—C20—N4—C21	174.2 (3)
C11—C10—C18—C8	173.3 (2)	N3—C20—N4—C21	−6.7 (4)

### Hydrogen-bond geometry (Å, °)

**Table:**

*D*—H···*A*	*D*—H	H···*A*	*D*···*A*	*D*—H···*A*
N1—H1B···O2^i^	0.80 (5)	2.39 (5)	3.004 (3)	135 (4)
N2—H2B···O3^ii^	0.77 (4)	2.32 (3)	3.065 (4)	164 (3)
N3—H3···O1^i^	0.82 (4)	2.54 (4)	3.181 (3)	136 (3)
N4—H4D···O1^iii^	0.87 (4)	1.92 (4)	2.785 (3)	174 (3)
C4—H4B···Cg4^iv^	0.96	3.02	3.721 (3)	131
C10—H10···Cg4^v^	0.98	3.11	3.914 (3)	141

Symmetry codes: (i) −*x*+2, −*y*+1, −*z*+1; (ii) *x*+1/2, −*y*+3/2, *z*+1/2; (iii) *x*−1/2, −*y*+3/2, *z*−1/2; (iv) −*x*+3/2, *y*−1/2, −*z*+1/2; (v) −*x*+1, −*y*+1, −*z*.
